# Optimization of Sampling Positions for Measuring Ventilation Rates in Naturally Ventilated Buildings Using Tracer Gas

**DOI:** 10.3390/s120911966

**Published:** 2012-08-30

**Authors:** Xiong Shen, Chao Zong, Guoqiang Zhang

**Affiliations:** Department of Engineering, Faculty Sciences and Technology, University of Aarhus, Blichers Allé 20, Tjele 8830, Denmark

**Keywords:** response surface methodology, optimal design, desirability, sampling positions, tracer gas

## Abstract

Finding out the optimal sampling positions for measurement of ventilation rates in a naturally ventilated building using tracer gas is a challenge. Affected by the wind and the opening status, the representative positions inside the building may change dynamically at any time. An optimization procedure using the Response Surface Methodology (RSM) was conducted. In this method, the concentration field inside the building was estimated by a three-order RSM polynomial model. The experimental sampling positions to develop the model were chosen from the cross-section area of a pitched-roof building. The Optimal Design method which can decrease the bias of the model was adopted to select these sampling positions. Experiments with a scale model building were conducted in a wind tunnel to achieve observed values of those positions. Finally, the models in different cases of opening states and wind conditions were established and the optimum sampling position was obtained with a desirability level up to 92% inside the model building. The optimization was further confirmed by another round of experiments.

## 1. Introduction

The ventilation rate is a crucial parameter for controlling the indoor climate of buildings. It can affect the temperature, humidity and gas concentration around occupants, which is very important for their living or working conditions [1,2]. High concentrations of polluted gases, uncomfortable temperatures and humidity conditions harm their emotional and physical health [3]. Moreover, the resulting emissions of ammonia and odor from livestock production buildings may cause harmful impacts on the neighboring atmospheric environment and the surrounding citizens [4].

However, determining the actual ventilation rate in a naturally ventilated space with large openings is always a challenge. Tracer gas experiments are commonly conducted to calculate the ventilation rate from the difference between indoor and outdoor concentrations. The tracer gas method prefers the good mixing of tracer gas inside the building. For those buildings, imperfect mixing would lead to large uncertainty of the resulting ventilation rate measurements. It is very difficult to achieve in naturally ventilated buildings [5]. In this context, it is very important to find the optimal sampling position for the indoor concentrations.

Thus, research is needed for decreasing the sampling positions and finding out the optimal regions in order to estimate the ventilation rate. Buggenhout *et al.* tested 36 indoor tracer gas sampling positions to find the optimal ones for the ventilation rate using the decay method. They concluded that the measurement errors can be as large as 86% of the actual ventilation rate by choosing an improper sampling position. The outlet position which gave measurement errors below 10% was found to be optimal. This study was conducted in mechanically ventilated livestock buildings. Unfortunately, little literature can be referred to for determining the optimal sampling positions in naturally ventilated building [5]. The optimal sampling position of exhaust air in naturally ventilated buildings is very difficult to find, and involves large uncertainty. This is either because the air outlet of a natural ventilation system may shift with varied wind conditions and opening states, or the inlet and outlet can exist simultaneously at one opening [6–8]. At present, measurement in such a building requires a lot of sampling positions and expensive multi-point measurements and it is very time consuming. A hypothesis is that the measured values at the optimal sampling positions are representatives for the mean value of the whole-field indoor concentration [9]. Hence, many scientists have measured several sampling positions inside the building and calculated the average to represent the indoor concentration [5,8]. Due to the non-perfect mixing, large uncertainty still lies in their methods [5].

Another difficulty is that the optimal sampling positions may change location when the opening states are modified or the wind direction changes. This is a challenge for us when intending to measure the indoor concentration by using fewer sampling positions. Therefore, it is necessary to determine the sampling positions independent of the opening states and wind directions.

The objective of this study is to find the optimal sampling positions for ventilation rate measurement in a naturally ventilated building. In this research, we focused on the tracer gas measurement method. The investigation includes eight different cases, with four opening states and two wind directions in perpendicular or oblique wind directions. The concentration fields of the tracer gas were modeled with spatial variables and the optimal positions were determined.

## 2. Experimental Facility

### 2.1. Wind Tunnel and Scale Model Building

**Figure 1.**
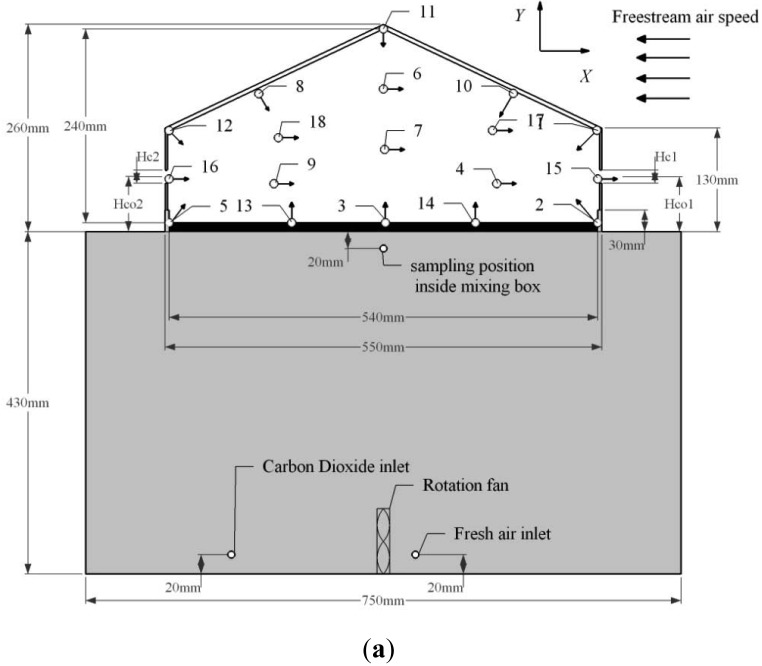

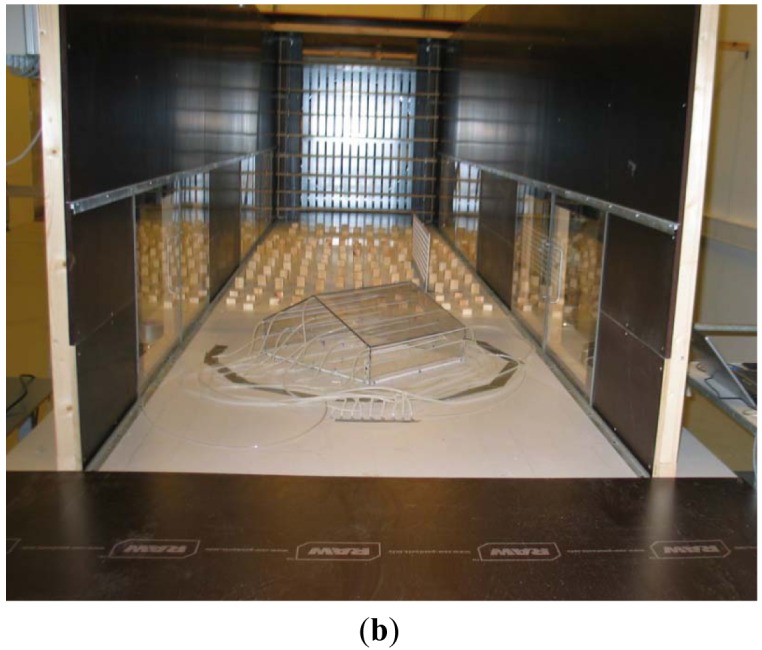
(**a**) Layout of sampling positions for measuring the indoor tracer gas concentration at the cross-section plane of the scaled building. (○) represent the sampling positions and 1–18 are labels of the positions, and the arrows indicate the direction of sampling's opening against the airflow in the room. *X,Y* is the coordinate of the plane and the origin point lies in the middle of the floor. *Hc_1_, Hc_2_* represent the windward and leeward opening size, respectively; *Hco_1_, Hco_2_* represent the height from the center of windward and leeward opening to the floor, respectively. (**b**) Connection of sampling pipes in wind tunnel.

The experiments were performed in a wind tunnel located in the Air Physics Lab, Aarhus University, using a model building. Figure 1 shows an experimental setup in the wind tunnel. The wind tunnel has double sidewalls and the ceiling surface was removed at the working section. The working section had a dimension of 6.00 m (length) × 1.38 m (width) × 1.55 m (height). 107 wooden blocks with dimensions of 50 mm × 50 mm × 50 mm and 25 mm × 50 mm × 50 mm respectively, were placed in the front of the working section with a distance of 100 mm apart in between to create an amount of roughness on the floor and to make the velocity profile at the working section similar to the natural wind over agricultural fields. The resulting reference wind velocity at 0.5 m high above the floor was equal to 3.2 m/s. and the wind profile can be described in [10]:

(1) $$\begin{array}{cc} v=(3.46\pm 0.02)h^{0.17\pm 0.004} & \left({\text{R}}^{2}=0.97\right) \end{array}$$

where, *v*, air velocity, m/s and *h*, height measured from the floor, m.

A 1:25 scale model building was represented as a sub-section of the stand-alone livestock building. The dimension and layout of the scale model building in the wind tunnel are shown in Figure 1. The roof and wall of the model building were made of a 5 mm thick clear acrylic sheet. The external dimensions of the models were 500 mm in length and 550 mm in width, and ridge height 260 mm. The sidewall under the eave was 130 mm high, and the resulting fully opening size was 100 mm. The roof slope was 25° and volume of the building was 0.11 m^3^. The internal space of the model building had the width of 270 mm, height of 240 mm and side eave height of 110 mm.

### 2.2. Tracer Gas Release and Measurement Technique

In order to measure the ventilation rate through the ventilated scale model building, the tracer gas constant injection method [11,12] was adopted in this study. The constant method was often used when the ventilation rate remained constant during the measurement period, similar as this study. In the beginning, the injection volume flow of tracer gas was injected to the measured building space at a constant, *q*(m^3^/s). After the steady state of the indoor concentration field has been reached, concentration differences between the indoor and outdoor, *(C_i_* − *C_o_)*, were monitored simultaneously. The ventilation rate *Q* (m^3^/s) was then calculated by Equation (2):

(2) $$Q=q/(C_{i}-C_{o})=q/(C\cdot C_{o})$$

In this study, *C* is the dimensionless gas concentration, which is defined as:

(3) $$C=(C_{i}-C_{o})/C_{o}$$

where, *C_i_, C_o_* is the measured indoor and the outdoor concentration, respectively.

Setup of the tracer gas experiment is shown in Figure 1. CO_2_ was used as the tracer gas and released from a CO_2_ tank (27 L). The air current was guided into a gas regulator and a control valve in order to keep the CO_2_ injection rate stable at a constant level of *q* = 2.5 × 10^−6^ m^3^/s. The pure CO_2_ was then mixed with the room air in an air mixing box, where a rotation fan was placed at the bottom to enhance the air mixing. An air pump at the speed of 7.17 × 10^−5^ m^3^/s was used to deliver free air into the box to mix with the pure CO_2_. The top of the box was connected to the indoor space through approximately 100 orifices evenly distributed on the floor. The mixed air in the box can be delivered into the building space through these orifices.

The indoor and outdoor CO_2_ concentration was monitored and recorded by an INNOVA 1309 multiplier and analyzer. The sampling duration of each recording was 5 s and the flush time of the instrument chamber between two recordings was 20 s. Three such continuous recordings and flashings were taken for each sampling position.

### 2.3. Experimental Cases

Table 1 shows the dimension of openings in different cases of opening state and directions. As shown in Table 1, eight cases of opening states and external wind directions were investigated in the research. Through these cases, different airflow patterns and variable concentration distributions of indoor space can be obtained. During the experiment, the opening states of the building were operated by changing the solid curtain mounted on the sidewall openings. The size of the sidewall opening remained the same in all cases. However, the locations of the openings were different. Two wind directions were investigated, one was the perpendicular and the other was oblique direction. The directions were modified by rotating the scale model building around the center point on the wind tunnel platform as seen in Figure 1(b).

## 3. Theoretical Consideration: Response Surface Methodology

The Response Surface methodology aimed to formulate an approximated model to predict the response by the related variables and adopts for the optimization process [13]. Compared to other methods such as Artificial Neural Network (ANN) method, the RSM method has reported to be more efficient for the model establishment in cases that only few variables were involved [14,15].

Once we have successfully built up an approximate model to predict the concentration field, we can apply it to find out the optimal position in the field for measuring the ventilation rate. In this study, the concentration field can be predicted by the approximate mathematical model that is established by the RSM method. The underlying model has the form: *C* = *f(x,y)* + *ε*. where, C is the dimensionless concentration calculated by Equation 3. *(x,y)* is the dimensionless coordinate and obtained from the spatial coordinate *(X,Y)* divided by the height of building (260 mm). The coordinate of the field is shown in Figure 1(a) and the origin point lies in the middle of the floor. *ε* is the error term.

Normally, *f(x,y)* in the RSM method is expressed approximately by polynomial regression model [15]. The quadratic or third-order polynomial model is commonly used [13]. However, compared to the quadratic model, even the third-order model would increase more experimental loads, but would enhance the accuracy [16]. In order to achieve high accuracy, we adopted the third-order polynomial model instead, which can be expressed by Equation (4):

(4) $$f(x,y)=\beta_{0}+\beta_{1}\;x+\beta_{2}\;y+\beta_{3}\;x^{2}+\beta_{4}\;y^{2}+\beta_{5}\;xy+\beta_{6}\;xy^{2}+\beta_{7}\;x^{2}y+\beta_{8}\;x^{3}+\beta_{9}\;y^{3};g(x,y)\leq 0$$

The overall procedure of the RSM method involves following steps [15]:

- Designing the experiment for adequate and reliable measurement of the response of interest. It involves: (1) construct the design space, (2) determine the number of design points, (3) locate the design points by experimental design methods.
- Developing a polynomial model of the third order response surface with the best fittings.
- Finding the optimal set of experimental parameters that produce optimum value of response.

### 3.1. Construct the Design Space

In the beginning of designing the experiment, it is necessary to construct the design space, where the sampling positions are chosen. As seen in Figure 1(a), the cross-section area of the scale model building is enveloped by the roof and sidewall and can be depicted mathematically by a constrained design space:

(5) $$\begin{array}{c} (110\;\text{mm}-240\;\text{mm})/(270\;\text{mm}-0\;\text{mm})\;X+Y-240\;\text{mm}\leq 0;\\ (110\;\text{mm}-240\;\text{mm})/(-270\;\text{mm}-0\;\text{mm})\;X+Y-240\;\text{mm}\leq 0;\\ -270\;\text{mm}\leq X\leq 270\;\text{mm};\\ 0\;\text{mm}\leq Y \end{array}$$

In which, *X (mm), Y (mm)* is the spatial variables in height and width direction of the building, respectively; the origin point of the *X* − *Y* coordinate is the center of the floor. The dimensionless form of the constraint form is shown in Equation (6), where the spatial variables are normalized by the maximum height of the model building (260 mm).

In Equation (4), *g(x,y)* ≤ *0*, is the constraints form of the dimensionless coordinate*(x,y)*. As seen in Figure 1(a), the sampling positions should be chosen from the internal cross-section area of a pitched-roof building. The cross-section area was constructed by the building envelop, which can be expressed mathematically by:

(6) $$\begin{array}{c} -27y+13x\leq 24.9231;\\ 27y+13x\leq 24.9231;\\ -1.04\leq x\leq 1.04;\\ 0\leq y \end{array}$$

### 3.2. How to Locate the Design Points: Optimal Design

From the constraints form [Equations (5) and (6)], a design space was constructed, within which the sampling positions for experiment can be chosen. In order to formulate an appropriate model that represents the concentration field of the tracer gas, attention should be paid to the choice of the sampling positions to building up the model. The model accuracy would decrease if there are not sufficient sampling positions within the design space. Moreover, the efficiency of those positions for the model establishment is not verified [15]. Thus, several experimental design methods can be developed to select the sampling positions, such as the factorial design, orthogonal design, central composite design, and optimal design, *etc.* [17].

The prediction by the established model may involve certain variance compared with the observed response. The variance is found to be related with the chosen experimental design methods [16]. The methods with less variance and larger robustness are preferred for the model establishment. However, among those experimental design methods, the optimal design is mostly suitable for this study. That is either because only it can function well when the design space is constrained as similar as this study. Or it can establish the model with low bias and minimum-variance [15].

### 3.3. IV Optimality and Distance Based Design

The optimal design method can find out the sampling positions based on the specified optimality criterion [15]. The basic principle of the optimality criterion is to decrease the model variance and bias to the minimum level. In this study, the IV-optimal design/or IV-optimality was used as the criterion. It sought a way to find the sampling positions with minimum integral prediction variance across the design space. Consequently the model can be built to provide lower prediction variance and less bias [16]. Detailed information of this method and the optimal design can also be found in the literatures [16,18].

Despite the reduction of model bias, the sampling positions should be well distributed in the design space. Thus the “space filling design”, is crucial and should be followed to show extensive information of the overall design space. However, the IV-optimality may not be efficient to achieve that because it searches the sampling that can only decrease the model variance but not consider the spread within the design space. In this study, the Distance based design is often coupled with IV-optimal design to make the sampling positions full filling in the design space [19]. That is because the Distance based design chooses sampling positions in a way that achieves maximum spread throughout the design region.

Thus, as the first step, the IV-optimal design method was adopted to search the design space to find out efficient sampling positions to build up the model. And then the Distance based design fill the gap between those existing positions and make the design space “space filling”. This method had reported to use in some researches in term of its comprehensive and redundant characteristics [15,20–23].

### 3.4. Number of Sampling Positions

#### 3.4.1. Minimum Required Number of Sampling Positions

In order to determine the number of sampling positions to develop the model in Equation (4), it is crucial to calculate the minimum number *P* of the sampling positions [Equation (7)], which depends on the degree of freedom of the model terms:

(7) P=(d+k)!/d!k!

where, e.g., *d* = *2* is the order of the regression model, *k* = *2* is the number of design variables. Thus, the resulting minimum number of sampling positions *P* = 6. And *d* = *3, k* = *2, P* = *10*.

#### 3.4.2. Total Number of Sampling Positions

The total number of sampling positions that necessarily required constructing a regression model was determined by the experimental design tool contained in the Design Expert ver. 8 software. The design tool is named as the Fraction of Design Space (FDS), which aims to evaluate the efficiency of the chosen number of sampling positions, as well as the quality of design method. In order to do that, this tool will observe what percentage of the design space is under the actual experimental error based on the expected variance of the response at certain statistically significance level. And thus, the expected variance of the response and the actual experimental error was necessary to be known [21,24–26].

The expected change of the response detected by the established model (*C*) was determined by the minimum measurement error of the ventilation rate, *Q*.

According to Equations (2) and (3), the absolute relative error of *Q* was calculated by propagation of error from the recorded variables [27]:

(8) $$\sigma Q/Q=\sigma (q/((C\cdots C_{o}))$$

where *σQ, σC_i_, σC_o_* was the experimental error of the ventilation rate, local indoor concentration, and outdoor reference concentration, respectively. Based on preliminary experiment, the fluctuation of outdoor reference concentration *σC_o_/C_o_* was very small (≤*0.01*), thus we assume *σC_o_* = *0*. The tracer gas injection speed *q* was maintained constant and Equation (8) becomes:

(9) σQ/Q=σC/C

The measurement uncertainty of *Q* was expected to be in the range of 0.15∼0.40 for natural ventilated buildings according to previous studies [5]. Based on this, the expected relative variance of the ventilation rate, *σQ/Q*, was defined as 0.15 in this study.

Thus according to Equation (9), the minimum change of the response *C* detected by the RSM model was equal to 0.15. Since the actual experimental error was unknown prior to the designed experiment, a preliminary experiment was necessary for obtaining the approximate error value. The setup of the wind tunnel, scaled model building and background concentration (*C_o_*) measurement can be seen in Section 2.1 of this paper. And the indoor concentration, *C_i_*, was measured at the outlet of the model building. Based on Equation (3), the experimental error (*σC/C*) was calculated by:

(10) $$\sigma C/C=\sigma ((C_{i}/C_{o})/(C_{i}/C_{o}-1))=\sigma C_{i}/(C_{i}-C_{o})$$

*C_o_, C_i_* and *σC_i_* are the value of mean outdoor concentration, mean and standard deviation error of indoor concentration. The measurement of the indoor and outdoor concentration was repeatedly recorded at time duration of two hours. Calculated by Equation (10), the value of experimental error *σC/C* was equal to 0.087.

Based on the minimum expected change of the response and the estimated experimental error, the Design Expert ver. 8 software can predict the distribution of standard error in the design space. The distribution of standard error was modeled by the sampling positions based on the estimated third-order model as shown in Equation (4). From the distribution, we can know the fraction of design space below the estimated experimental error (*σ* = *0.0866*) according to the statistically significance level (*p* = *0.05*). Large fraction of design space below the experimental error would be yielded if sufficient and efficient sampling positions being chose for the experimental design.

**Figure 2.**
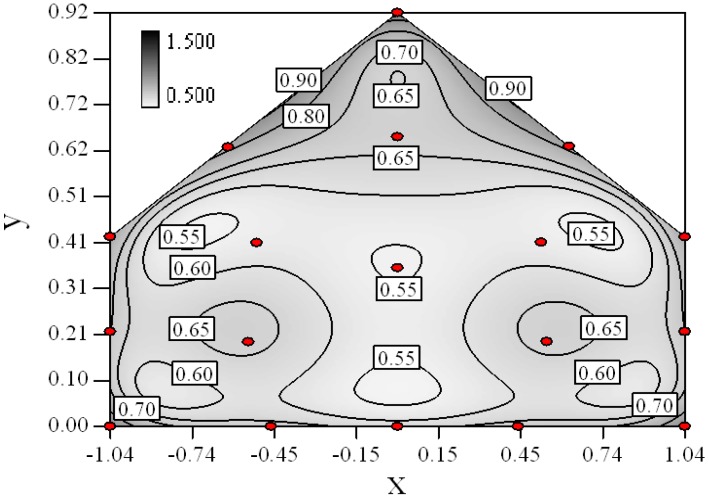
Distribution of standard deviation error as a third-order response surface from the selected sampling positions designed by IV optimal design. The minimum detectable change in the response is 0.15, mean experimental error is 0.087, and significant level is 0.05. Within the design space, 94% section of region area is lower than the surface-averaged standard error, which is equal to 0.75.

Figure 2 shows the distribution of the standard error with 18 sampling positions. As we defined 18 sampling positions, we can see from Figure 2 that 94% of the design space was below the experimental error, which is superior to the recommendation of 80% [15]. This indicates the good quality of the chosen number of sampling positions and the experimental design method. In addition, those sampling positions spread thoroughly in the design space and every joint vortex of the boundary lines and indicted that the design was space-filling.

It can also be found that large standard error near the boundary of the design space from Figure 2, which means the measurement should be carefully conducted or repeated in order to decrease the model variance in these regions.

### 3.5. Model Development

Followed by design of experiment and conduct of the designed experiment, the corresponding three-order RSM model was established respectively with the general form as depicted in Equation (4) based on the measured data of the response. In order to examine the fitting of the model, such indexes as *Adjusted R^2^* and *Predicted R^2^* were used in our study [15].

*Adjusted R^2^* is the *R^2^* adjusted by the number of terms in the model, where, *R^2^* is a measurement of the amount of variation around the mean explained by the model:

(11) $$R^{2}=1-({\text{SS}}_{\text{error}}/{\text{SS}}_{\text{Total}})$$

*SS_error_* is the sum of squares of residuals; *SS_Total_* is the total sum of squares.

(12) $$\text{Adjusted}\;R^{2}=1-({\text{SS}}_{\text{error}}/{\text{SS}}_{\text{Total}})({\text{df}}_{\text{Total}}/{\text{df}}_{\text{error}})=1-({\text{SS}}_{\text{error}}/{\text{SS}}_{\text{Total}})((N-1)/(N-p))$$

where, *df_Total_, df_error_* is the degrees of freedom, which are equal to *N* − *1, N* − *p* respectively. *N, p* is the number of sampling positions and model terms. The established models are compared by the *Adjusted R^2^*, not by the *R^2^*, considering their number of terms, *P*, was varied.

*Predicted R^2^* is also a measure of how good the model predicts a response value. It is computed as:

(13) $$\text{Predicated}\;R^{2}=1-(\text{PRESS}/({\text{SS}}_{\text{Total}}))$$

where, Predicted Residual Error Sum of Squares (*PRESS*) is a measure of how the model fits each point in the design. *PRESS* is calculated by systematically removing each observation from the data set, estimating the regression equation, and determining how well the newly established equation predicts the removed observation. The sum of squares between the observations and predictions are then summed to calculate the value of *PRESS*.

Of all cases, the established model that presented the low *Adjusted R^2^* and *Predicted R^2^* were required to be enhanced [28]. Thus in this study, backward regression method [21] was utilized to revise the established model by eliminating the non-significant terms. In the beginning of the regression process, the full model was established with the form as shown in Equation (4). We pre-defined the threshold value *p* equals to 0.10, and those terms lower than this *p* value would be remained. The elimination of terms of the model would terminate until most of them can satisfy the *p*-value. By this method, each term of the full model will give a chance to be included in the model and thus the model can be enhanced.

Transformation of the response is an important method when the model is formulated with poor fitting to the observed response [15]. The quality of fitting can be seen from how the errors (residuals) of the model go with the magnitude of the response (predicted values). Box-Cox Transformation [15] is a commonly applied transformation method, especially when the residuals are not stabilized and the statistical distribution does not present normal distribution. In this study, we evaluated square Root, natural log, inverse square root and inverse *etc.* of transformation function to enhance the established model. After the backward regression and Box-Cox Transformation, the RSM model with best quality of fit with the observations would be used for the optimization process.

### 3.6. Optimization

The desirability function [13,29] was established based on the RSM model and used for optimization process. It made use of an objective function, which depend on the target goal defined by the users.

The objective function reflects the level of desirability. When the observation is close to the target, the desirability level is high. The range of the desirability was from zero to one (minimum to maximum desirability, respectively).

In this study, for each case as listed in Table 1, the object function *d_i_* (*1* ≤ *i* ≤ *8, i ∊ N*) came from the according RSM model. The simultaneous objective function, which showed the overall disability distribution within the design space, was equal to a geometric mean of all cases:

(14) $$D={\left(d_{1}\times d_{2}\times \ldots \times d_{8}\right)}^{1/8}$$

and *d_i_(Ĉ_i_)* was defined as:

(15) $$\begin{array}{c} {\hat{C}}_{i}(x,y)<L_{i},d_{i}({\hat{C}}_{i})=0;\\ L_{i}\leq {\hat{C}}_{i}(x,y)\leq T_{i},d_{i}({\hat{C}}_{i})={\left(\frac{{\hat{C}}_{i}(x,y)-L_{i}}{T_{i}-L_{i}}\right)}^{s};\\ T_{i}\leq {\hat{C}}_{i}(x,y)\leq U_{i},d_{i}({\hat{C}}_{i})={\left(\frac{{\hat{C}}_{i}(x,y)-U_{i}}{T_{i}-U_{i}}\right)}^{t};\\ {\hat{C}}_{i}(x,y)>U_{i},d_{i}({\hat{C}}_{i})=0 \end{array}$$

where, *Ĉ_i_, Ĉ_i_(x,y)* was the estimated concentration ratio by the established RSM model. And *L_i_, T_i_* and *U_i_* was the low limit, target value and upper limit of *Ĉ_i_(x,y)* of each case, respectively. The exponent *s* and *t* represented the degree of importance to hit the target value, respectively. In this study, both values equal 1 because of the same importance as the values getting close to the target value. If any of the responses or factors is not in their desirability range, the overall function becomes zero.

Our goal was to find the optimal sampling positions for the tracer gas measurement; the goal can be determined as the target value, maximum, minimum or a range within the design space [15]. For simultaneous optimization, the response of each case is assigned a low, high limit value and a target. In our study, we set the target zone of each case where the local concentration was close to the surface average:

(16) $$\frac{\iint_{S}f(x,y)\text{dxdy}}{\iint_{S}\text{dxdy}}$$

where, *S* is the constrained region by spatial variables *x* and *y. f(x,y)* is the estimated response by the model regressed from the observed experimental data. Finally, the optimal position was confirmed by another new experiment, and the desirability and observed response values were compared with the estimation by the regression model.

## 4. Sampling Positions in Wind Tunnel Experiment

Layout of sampling positions for the indoor CO_2_ concentration that obtained from the RSM method is given in Figure 1(a) and detailed information is given in Table 2.

The sampling position of the background concentration was located 0.6 m upstream of the scale model building, 0.1 m above the wind tunnel floor. The gas concentration inside the air mixing box was measured close to the top surface of the air mixing box as seen in Figure 1.

Sampling pipes with a diameter of 3 mm were used to take air samples in all positions. These sampling pipes were mounted on the building endwall and horizontally placed along the building length. Eight sampling orifices with dimensions of 1 mm were evenly distributed along each pipe with an interval of 50 mm. The indoor air at a sampling position was sucked from two ends of the pipe and delivered to the multiplier and the gas monitor through the external pump (230 V, 6.3 × 10^−5^ m^3^/s).

As seen from Figure 2, the sampling positions at the border of the design space contain larger standard error as predicted the third-order response surface model. Thus, during the experiment, observations at all sampling positions were recorded continually at long time duration of 2.5–4 h after the CO_2_ concentration inside the mixing box came to a constant.

## 5. Result and Discussion

### 5.1. Measurement Result

Table 3 shows the direct measurement results of all cases (Cases 1–8) as listed in Tables 1 and 2. For all the cases, the relative error of the concentration measurement is lower than 8%, which shows a reasonable experimental error. The relative error of the position in the mixing box is below 0.5%, which indicates a stable source of tracer gas during the experiment. The direct measurement results are then turned into dimensionless results by Equation (3).

### 5.2. Model Establishment

Table 4 shows the established models in all cases of opening states and wind directions. The actual responses of the model are the dimensionless concentration, *C*. The RSM models in Table 4 are different from the full model as shown in Equation (4) because they are reduced from the full regression model by eliminating the non-significant terms within it. Meanwhile, the observed responses are enhanced by using transformation functions such as natural log and square root.

When the *Adjusted R^2^* is closer to 1, the scattered sampling positions in the actual-predicted plot would accumulate in a line, indicates the predicted values are well in accordance with the actual values. It is noted that high *Adjusted R^2^* and *Predicted R^2^* of an established model do not occur simultaneously. As the degree of polynomial increases, the *Adjusted R^2^* will increase; however, it is not always the same for the *Predicted R^2^*. Even a high order polynomial function can obtain a high *Adjusted R^2^*, but may suffer from the low *Predicted R^2^*. Thus, in order to establish an accurate model, it is necessary not just to focus on the *Adjusted R^2^* solely, but also consider the *Predicted R^2^* comprehensively. That is because the latter is also crucial to determine the quality of an established model. It is reported that the difference between *Adjusted R^2^* and *Predicted R^2^* should be within 0.2 [28]. From Table 4, six of the eight cases are in accordance with this requirement. Even though Case 5 and Case 6 do not fulfill it, however, both established models show relative high and positive *Predicted R^2^* and *Adjusted R^2^*. This indicts that the models in Case 5 and 6 would be somehow efficient among all cases and can be adapted.

### 5.3. Predicted Concentration Field

Contour plots of the established RSM models predicting the concentration ratio are shown in Table 5.

As we see from Table 5, the concentration field varies between cases because of different opening states and wind direction (Table 2). For Cases 1–4, the wind direction is perpendicular to the building sidewall. As seen in Table 5, the concentration ratio is low behind the windward opening, particularly with the opening close to the floor. High concentrations are found in the region close to the floor and near the leeward sidewall. With the windward opening in its high position the high concentrations are found near the floor under the opening. This phenomenon also exists in Cases 5–8, in which the incoming wind direction is 45° to the sidewall. It may indicate that location of the windward sidewall opening effects the distribution of response. Besides, Case 3 and Case 6 which have different wind direction and the same location of opening, show that the wind direction effects the distribution.

### 5.4. Optimization

Table 6 shows the distribution of desirability for concentration measurement of each case. The desirable sampling positions should be representative and equal the volume average. Those red zones with the desirability close to 1 indicate that they are good and the blue zones close to 0 are bad for sampling positions. In separated cases, there are a big amount of candidate positions suitable for the measurement within the indoor space.

Thus, the objective function shown in Equation 15 may be utilized to calculate the geometric mean of desirability of all cases. Through this, we can obtain this adaptable function to predict the optimal positions for all cases.

**Figure 3.**
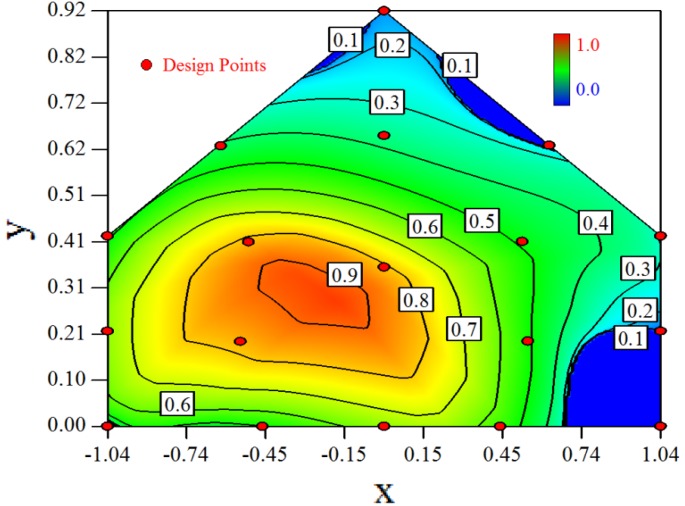
Desirability graph for the optimization of the concentration ratio within the design space, in which the optimal position = 0.93 shows the optimal position for the concentration measurement of all cases (Cases 1–8). The coordinates of this position are *x* = −*0.2, y* = *0.27*.

Figure 3 shows the desirability graph of all cases for the optimization of sampling position selection. The integrated desirability distributes as a convex surface with the spatial variables. The red zone lies in the leeward part of the indoor space and the maximum desirability equals 0.93 lies in the position of *x* = −*0.2, y* = *0.27*.

### 5.5. Confirmation of Optimal Selection

For validation purposes, the optimal sampling position obtained for all cases of opening states was tested by an additional new confirmation experiment. Table 7 presents the result of the confirmation test at the sampling position that shows the maximum desirability (*x* = −*0.2, y* = *0.27*).

In each case, the estimated concentration ratio of the optimal position is very close to the surface averaged concentration ratio and thus the estimated desirability is very close to 1. After the confirmation experiment, the measured concentration ratio of most cases is also quite close to the surface averaged and the actual desirability is close to 1, except for Case 4. Without considering Case 4, the actual integrated desirability of all other cases equals 0.86 and the estimated one is 0.92. Both are quite close to 1 which indicates the position is optimal for the indoor concentration measurement.

### 5.6. Further Discussion on the RSM Method

As seen from Table 4, the RSM model has shown desirable potential in predicting the concentration field. In Table 7, we can see that the optimal positions predicted by the model present high desirability for ventilation rate measurement. All of these can highlight the RSM modeling method in producing promising optimization and accurate modeling.

However, this method also shows some weakness and uncertainty in this study. In Table 7, we can find that Case 4 is distinct from other cases may because of the low estimated and actual concentration ratio in the optimal position. As seen in Equation (3), the low concentration ratio, *C*, may come from a low indoor and outdoor concentration difference, *C_i_* − *C_o_*. Besides, from Equation (10), if we kept the measurement error of the indoor concentration a constant, the relative error of *C* would be relative larger. These would make the actual response value difficult to detect in the experiment and thus lead to large uncertainty for Case 4.

In order to avoid the failure of such cases, two methods are suggested. One is to increase the amount of tracer gas released into the indoor space to increase the indoor concentration, and thus get a higher difference between the indoor and outdoor concentration. Another way is to decrease the experimental error of indoor concentration measurement.

A challenge of this method is to do a prior experiment before the designed experiment is done. The reason of doing this is because we have to know the actual experimental error, with which we can know how many sampling positions or design points we should define in order to overcome the expected model variance. This indicates that if more accurate prediction by the RSM method is expected, more sampling positions should be added into the design space to construct the model [14–16].

The advantage of this method is that it can be able to minimize the number of sampling positions and consequently decrease the workload on measurement. Buggenhout *et al.* divided the measured zone equally into 36 partitions and measured the concentration in each partition [5]. A linear interpolation performed between the measurements to visualize the spatial distribution. However, compared to the method, the sampling method for RSM modeling can be more efficient and statistically supported [15,17,23]. Moreover, this research investigated the concentration field with 3D spatial variables and was different from 2D in our study. Further research is recommended for 3D cases based on the RSM method.

Challenges also exist in finding the proper experimental design method to seek the sampling positions or design points in the design space. When the design space is regular and not constrained such as rectangular space, a lot of potential methods can be used, such as central composite design and factorial design [16]. However, in terms of constrained design space as this study, many classified design methods such as central composite design, full factorial design cannot be applied. In this context, optimal design method can be used to build up the model. This research use IV Optimality combined with Distance Based Design for sampling. Further study can be focused on other optimal design method in varied optimality criterion, such as D-optimality, A-optimality and so on.

## 6. Conclusions

This study used Response Surface Methodology (RSM) to determine the optimal sampling positions for the indoor concentration on the purpose to calculate the ventilation rate accurately.

1. The RSM modeling technique can be used to optimize the selection of sampling positions for environmental measurement. The target of the optimization was set to be the indoor space average. The optimal sampling positions for the indoor concentration measurement can be seen in the desirability graphs as shown in Table 6. The universal optimal sampling positions of all cases found to lie in the region as shown in Figure 3. The desirable region is recommended to be adopted for the sampling of indoor gas concentration in future experiment. Further study is recommended for such an aspect. But cautions should be taken as this method may suffer from inadequate estimation because weak observed values may easily fall into the experimental error.
2. Optimal design method was used to search for the design points in a constrained two dimensional design space. The design space mathematically represented a cross-section plane of indoor space in a naturally ventilated building. Thus, this method can be introduced for a naturally ventilated building to searching for the optimal position to measure or monitor several environmental parameters, not only for gas concentration, but also for odor, temperature and humidity, *etc.*
3. The required number of design points by RSM to formulate the model is direct proportional to the expected error. In this study, we expected 15% uncertainty of the observed value and managed to formulate a three degree polynomial function. Eighteen design points were selected and the estimated error of 94% fraction in design space was under the actual experimental error.
4. Statistical tools such as backwards regression and Box-Cox transformation were utilized for model establishment in this research. The *Adjusted R^2^* and *Predicted R^2^* were used as indexes to show the goodness of fitness of the established model. Comprehensive consideration of two parameters should be made in the process of modeling.

## Figures and Tables

**Table 1.** Experimental setup of different cases (cases 1–8). *Hc_1_, Hc_2_* represent the windward and leeward opening size, respectively; *Hco_1_, Hco_2_* represent the height from the center of windward and leeward opening to the floor, respectively. *θ* is the angle between the direction of wind and the roof ridge of the model building.

**Case**	***Hc**_1_*	***Hc**_2_*	***Hco**_1_*	***Hco**_2_*	***θ***
	mm	mm	mm	mm	°
1	50	50	105	105	90
2	50	50	55	105	90
3	50	50	105	55	90
4	50	50	55	55	90
5	50	50	55	55	45
6	50	50	105	55	45
7	50	50	105	105	45
8	50	50	55	105	45

**Table 2.** List of sampling positions of tracer gas experiment, by IV optimal design from software Design Expert ver. 8. **Std** is the descending order of the standard error of the corresponding position. *x,y* and *X,Y* are the dimensionless and actual coordinates of the positions, respectively.

**Std**	**Position**	***x***	***y***	***X***	***Y***
8	1	1.04	0.42	270	110
3	2	1.04	0.00	270	0
2	3	0.00	0.00	0	0
5	4	0.54	0.19	140.4	49.1845
1	5	−1.04	0.00	−270	0
11	6	0.00	0.65	0	168
6	7	0.00	0.35	0	92
9	8	−0.61	0.62	−159.3	162
4	9	−0.54	0.19	−140.16	49.0287
10	10	0.62	0.62	161.244	162.364
12	11	0.00	0.92	0	240
7	12	−1.04	0.42	−270	110
14	13	0.44	0.00	113.4	0
15	14	1.03	0.21	267.3	54
16	15	−1.04	0.21	−270	55.0898
13	16	−0.46	0.00	−118.8	0
18	17	0.52	0.41	135	106.8
17	18	−0.51	0.41	−132.3	106.506

**Table 3.** Experimental result of all cases (Cases 1–8) listed in Table 2. AVG, STDEV and Rel.err represent the average, standard deviation and relative error of the recorded CO_2_ concentration within the sampling durations for different cases and positions. The Ref. and IMB represent concentration measurement position in front of building for reference and inside the air mixing box, respectively. Positions 1–12 are listed in details in Table 1.

**Position**	**Case 1**			**Case 2**			**Case 3**			**Case 4**		

	AVG (mg/m^3^)	STDEV (mg/m^3^)	Rel. err(%)	AVG (mg/m^3^)	STDEV (mg/m^3^)	Rel. err(%)	AVG (mg/m^3^)	STDEV (mg/m^3^)	Rel. err(%)	AVG (mg/m^3^)	STDEV (mg/m^3^)	Rel. err(%)
**1**	1,072.2	2.5	0.2	1,177.8	11.9	1.0	1,084.0	3.9	0.4	1,136.0	6.9	0.6
**2**	2,722.5	113.9	4.2	1,064.3	20.9	2.0	2,815.7	156.6	5.6	1,066.5	18.2	1.7
**3**	1,844.1	106.3	5.8	1,519.5	37.0	2.4	1,805.6	80.6	4.5	1,396.6	25.6	1.8
**4**	2,114.4	129.5	6.1	1,064.7	6.2	0.6	2,147.9	141.3	6.6	1,045.0	4.2	0.4
**5**	1,383.9	12.8	0.9	1,697.8	32.2	1.9	1,398.6	21.6	1.5	1,906.9	38.4	2.0
**6**	1,343.9	13.6	1.0	1,104.1	7.7	0.7	1,359.4	23.1	1.7	1,070.2	7.2	0.7
**7**	1,526.8	27.2	1.8	1,170.2	12.2	1.0	1,550.4	43.4	2.8	1,131.1	7.5	0.7
**8**	1,293.6	11.1	0.9	1,173.5	15.5	1.3	1,292.7	14.3	1.1	1,119.4	8.2	0.7
**9**	1,424.5	23.1	1.6	1,416.5	29.5	2.1	1,439.0	19.6	1.4	1,306.8	24.5	1.9
**10**	1,173.3	16.4	1.4	1,157.9	7.8	0.7	1,186.4	14.7	1.2	1,146.1	9.7	0.9
**11**	1,261.9	19.9	1.6	1,096.8	15.3	1.4	1,257.8	15.0	1.2	1,086.4	6.7	0.6
**12**	1,307.5	15.2	1.2	1,264.1	21.0	1.7	1,304.1	19.8	1.5	1,150.9	10.6	0.9
**13**	3,201.4	303.9	9.5	1,101.5	23.2	2.1	3,113.7	202.9	6.5	1,072.9	14.4	1.3
**14**	2,556.9	87.4	3.4	1,073.5	7.6	0.7	2,573.0	116.9	4.5	1,063.2	6.1	0.6
**15**	1,391.3	17.0	1.2	1,632.2	34.3	2.1	1,337.9	16.4	1.2	1,227.9	12.9	1.1
**16**	1,573.8	77.1	4.9	2,436.2	108.9	4.5	1,602.2	92.2	5.8	2,238.0	84.3	3.8
**17**	1,516.7	27.0	1.8	1,091.4	8.1	0.7	1,520.7	33.1	2.2	1,086.8	4.9	0.5
**18**	1,374.8	19.2	1.4	1,198.9	16.7	1.4	1,379.4	17.2	1.2	1,151.9	11.3	1.0
**Ref.**	1,006.2	3.5	0.3	1,013.9	8.2	0.8	1,018.8	3.3	0.3	1,021.3	2.9	0.3
**IMB**	47,770.8	246.3	0.5	49,327.1	139.7	0.3	48,670.9	128.8	0.3	47,981.8	73.3	0.2
**Position**	**Case 5**			**Case 6**			**Case 7**			**Case 8**		

**1**	1,213.1	15.8	1.3	1,098.9	7.5	0.7	1,086.4	17.8	1.6	1,253.1	14.9	1.2
**2**	1,196.6	26.7	2.2	2,215.1	118.4	5.3	2,264.5	151.4	6.7	1,211.4	33.7	2.8
**3**	2,437.4	182.9	7.5	2,650.9	188.7	7.1	2,869.2	197.4	6.9	2,549.0	186.8	7.3
**4**	1,165.2	16.5	1.4	1,867.3	76.0	4.1	1,905.9	76.3	4.0	1,183.6	22.5	1.9
**5**	1,736.0	40.1	2.3	1,534.2	36.8	2.4	1,420.5	26.9	1.9	1,630.2	47.3	2.9
**6**	1,214.4	30.4	2.5	1,621.3	31.3	1.9	1,686.8	28.4	1.7	1,320.4	51.4	3.9
**7**	1,371.9	25.9	1.9	1,812.8	39.5	2.2	1,879.7	44.3	2.4	1,514.0	68.4	4.5
**8**	1,200.4	15.5	1.3	1,473.3	17.3	1.2	1,501.3	18.6	1.2	1,240.7	18.3	1.5
**9**	1,776.5	63.2	3.6	1,646.2	47.3	2.9	1,738.1	54.5	3.1	1,980.9	61.9	3.1
**10**	1,192.3	14.9	1.2	1,489.8	26.9	1.8	1,525.5	27.9	1.8	1,248.5	26.1	2.1
**11**	1,127.3	20.8	1.8	1,459.1	20.2	1.4	1,502.0	23.4	1.6	1,154.7	18.9	1.6
**12**	1,265.5	37.0	2.9	1,410.5	13.1	0.9	1,481.1	12.1	0.8	1,382.0	27.1	2.0
**13**	1,602.3	84.6	5.3	2,767.6	148.3	5.4	2,793.0	111.7	4.0	1,641.1	93.1	5.7
**14**	1,038.2	14.0	1.3	1,886.3	116.0	6.1	2,015.3	109.3	5.4	1,038.7	10.1	1.0
**15**	1,374.0	14.5	1.1	1,452.8	14.8	1.0	1,483.0	19.9	1.3	1,725.7	39.2	2.3
**16**	3,080.9	165.2	5.4	3,779.8	273.1	7.2	4,092.6	236.9	5.8	3,614.4	222.3	6.2
**17**	1,177.0	24.6	2.1	1,599.2	36.3	2.3	1,655.9	34.0	2.1	1,250.2	33.0	2.6
**18**	1,380.9	42.2	3.1	1,613.7	27.1	1.7	1,672.6	46.8	2.8	1,534.0	39.0	2.5
**Ref.**	1,023.7	10.4	1.0	1,020.1	7.0	0.7	1,006.3	5.0	0.5	1,017.8	7.0	0.7
**IMB**	45,217.5	102.2	0.2	47,192.6	124.3	0.3	47,539.6	91.7	0.2	46,146.3	88.4	0.2

**Table 4.** Established RSM models and their quality of fit of varied cases (Case 1–8). Adj. R^2^ and Pred. R^2^ represent the *Adjusted R^2^* and the *Predicted R^2^* of the RSM model, respectively. In the Predicted vs. Actual plot, the prediction values(*y* axis) are calculated by the RSM model and the actual values(*x* axis) are the experimental results.

**Case**	**RSM model**	**Adj. R^2^**	**Pred. R^2^**	**Predicted *vs.* Actual plot**
Case 1	*f(x,y)* = *1.17057* + *0.85098x* − *2.46459y* − *1.84713xy* + *1.56661y^2^*	0.76	0.63	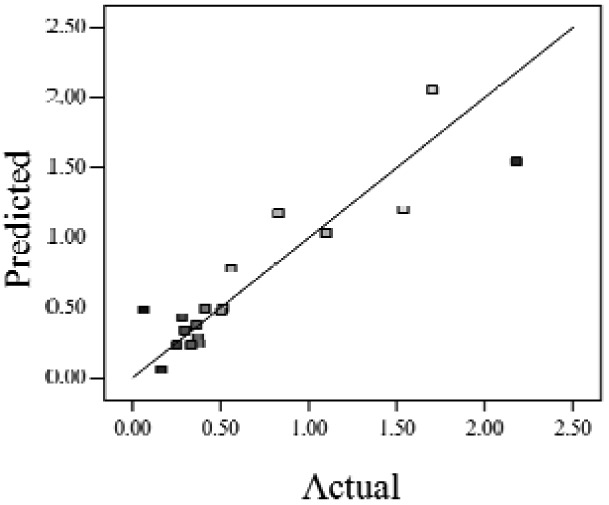
Case 2	*f(x,y)* = *exp(*−*0.78742* − *2.85369x* − *4.78776y* + *3.80147xy* − *1.19152x^2^* + *3.22112y^2^* + *4.39794x^2^y* + *0.95899x^3^)*	0.93	0.82	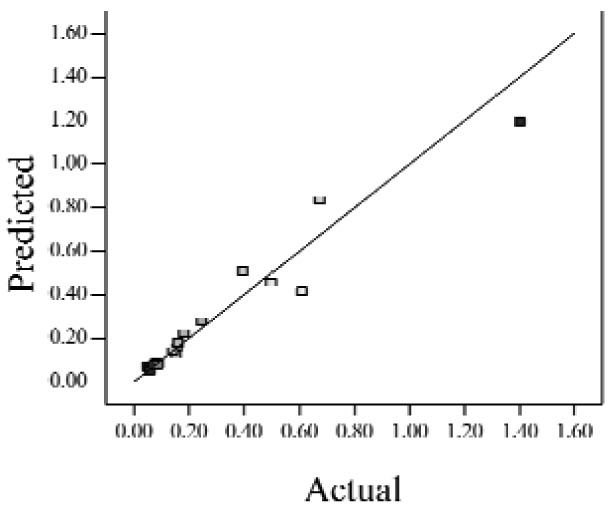
Case 3	*f(x,y)* = *(0.97401* + *0.45044x* − *0.75094y* − *1.04617xy) ^2^*	0.73	0.58	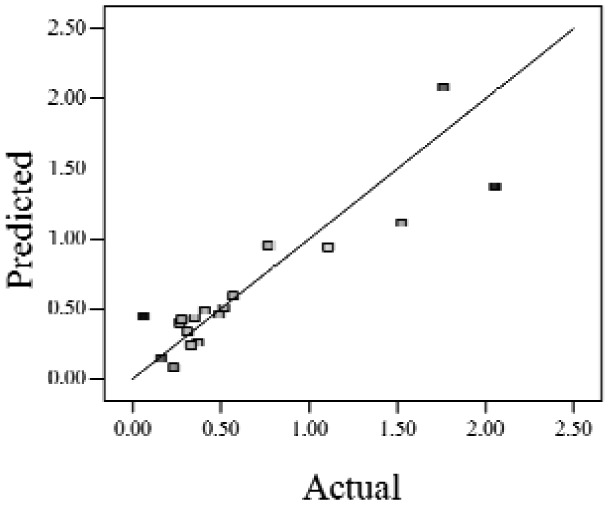
Case 4	*f(x,y)* = *exp(*−*1.71620* − *1.88740x* − *1.36309y* + *3.81367xy)*	0.67	0.54	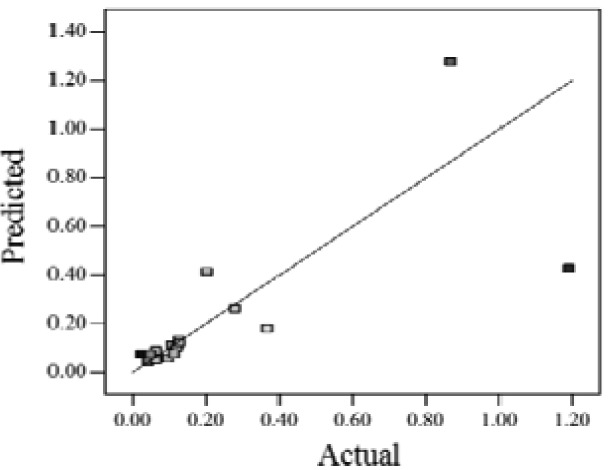
Case 5	*f(x,y)* = *exp(*−*0.058175* − *0.87132x* − *2.97033y* + *1.36252xy* − *0.72429x^2^* + *0.76924y^2^)*	0.75	0.52	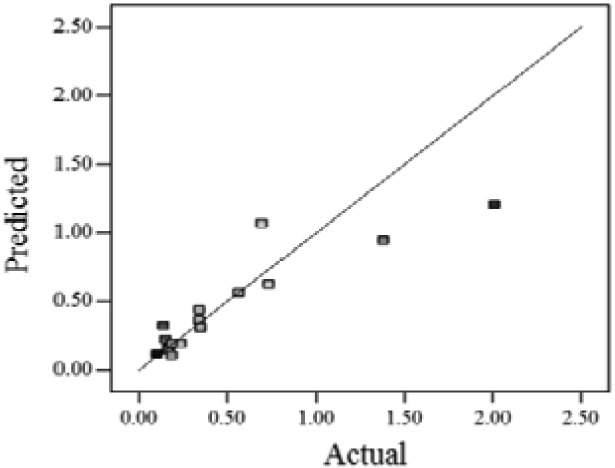
Case 6	*f(x,y)* = *(1.36578* + *0.026777x* − *1.63830y* − *0.35100x^2^* + *1.01114y^2^)^2^*	0.69	0.47	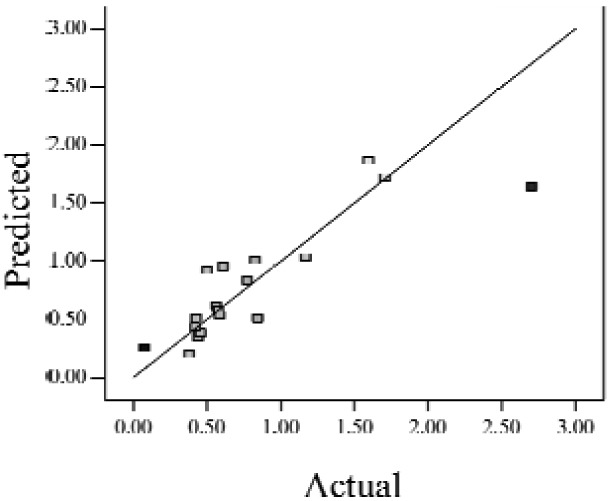
Case 7	*f(x,y)* = *(1.39195* + *0.030351x* − *0.90476y* − *0.43484x^2^)^2^*	0.59	0.40	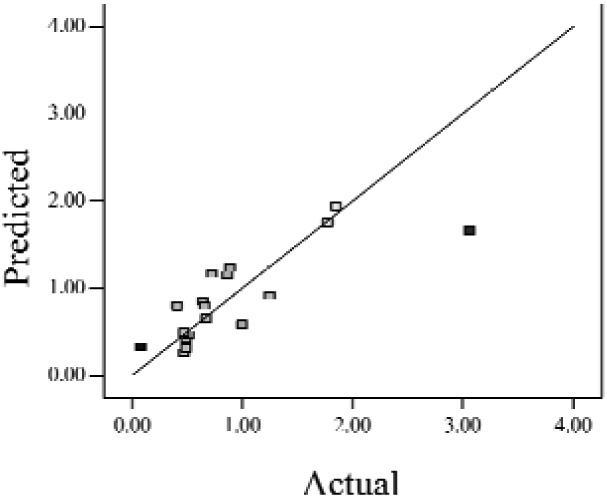
Case 8	*f(x,y)* = *exp(0.34953* − *1.69325x* − *4.00463y* − *1.97475xy* − *1.30496x^2^* + *1.67581y^2^* + *2.50985x^2^y* + *6.82542xy^2^* + *1.00282x^3^)*	0.90	0.78	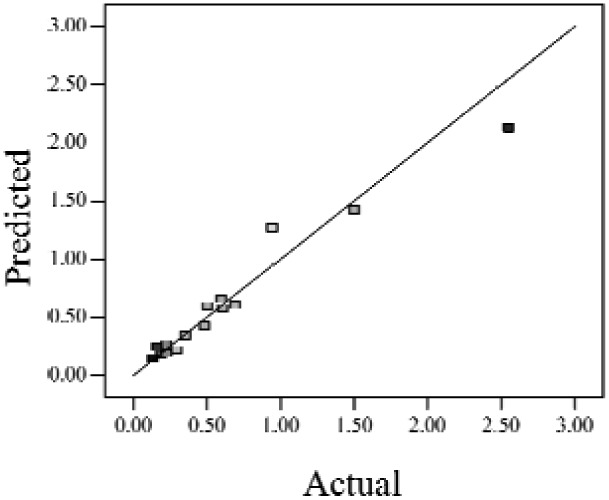

Actual values indicate the observations of the response.

**Table 5.** Contours of the response suface of the established enhanced regression model for different experiemental cases (Cases 1–8). The black bars in the plots represent the obstacles in the sidewall openings. In the plot, the incoming wind blows from the right side to the left.

**Response suface of the enhanced regression model in Cases 1–8**			
Case 1	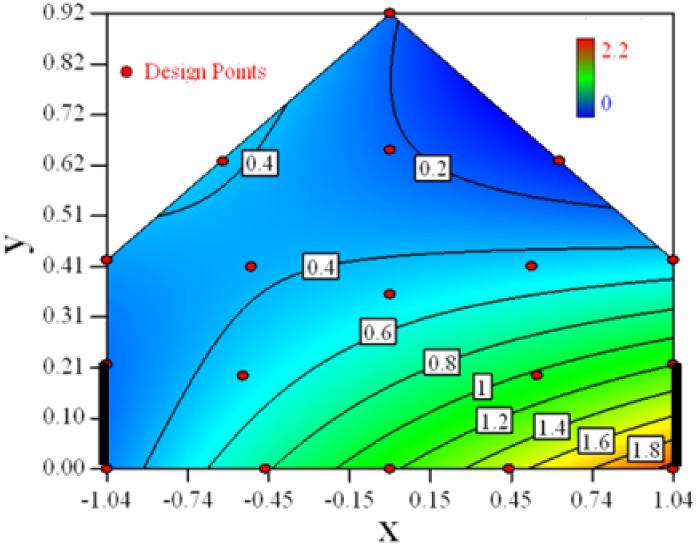	Case 7	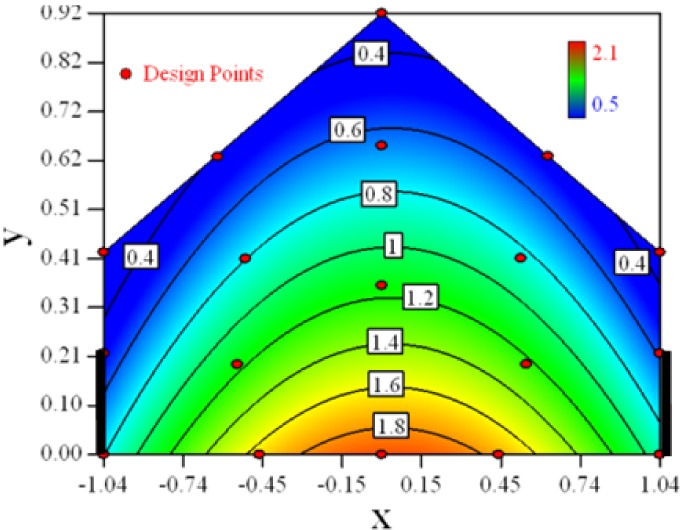
Case 2	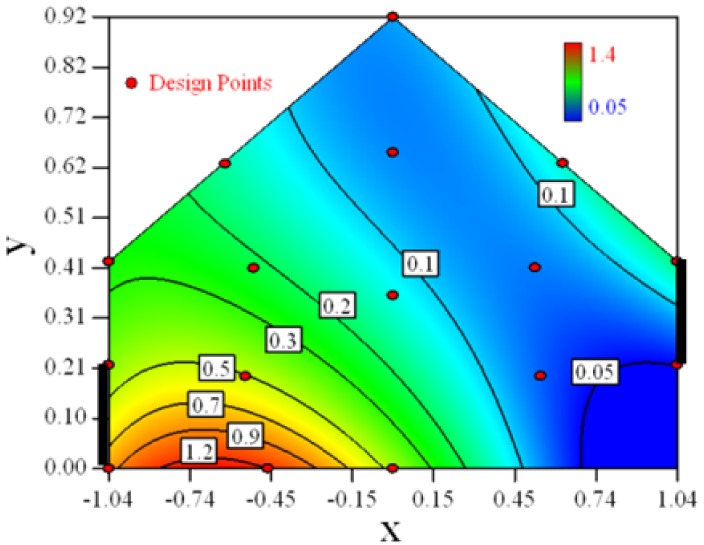	Case 8	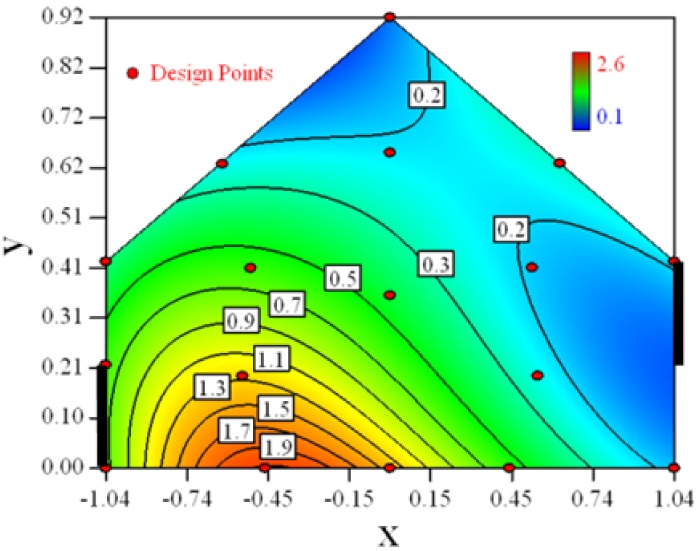
Case 3	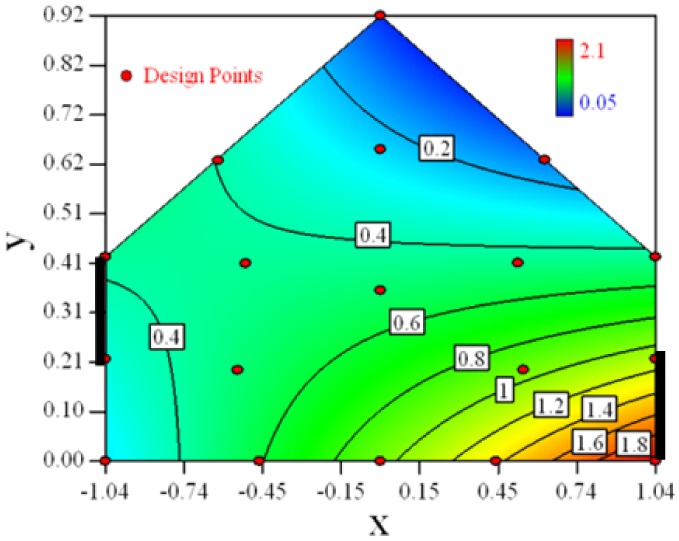	Case 6	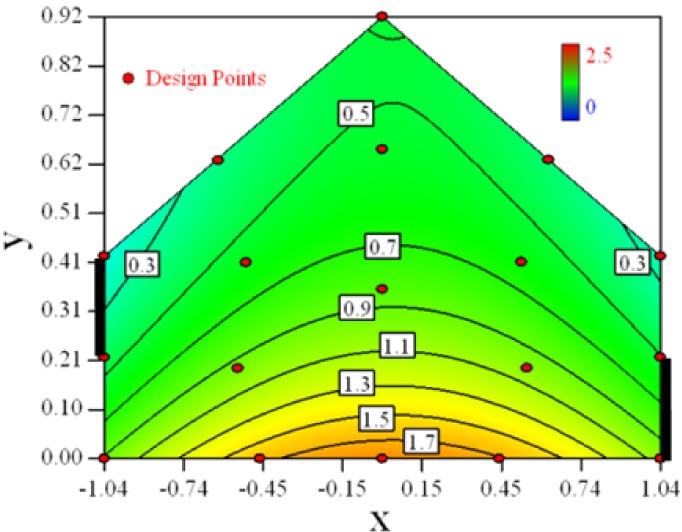
Case 4	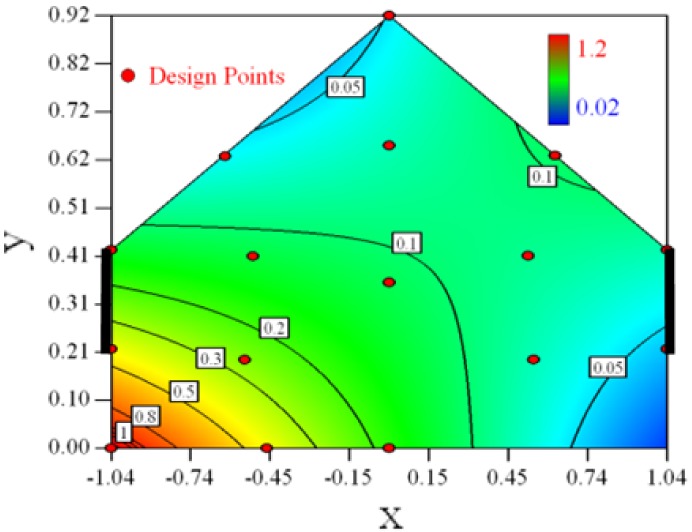	Case 5	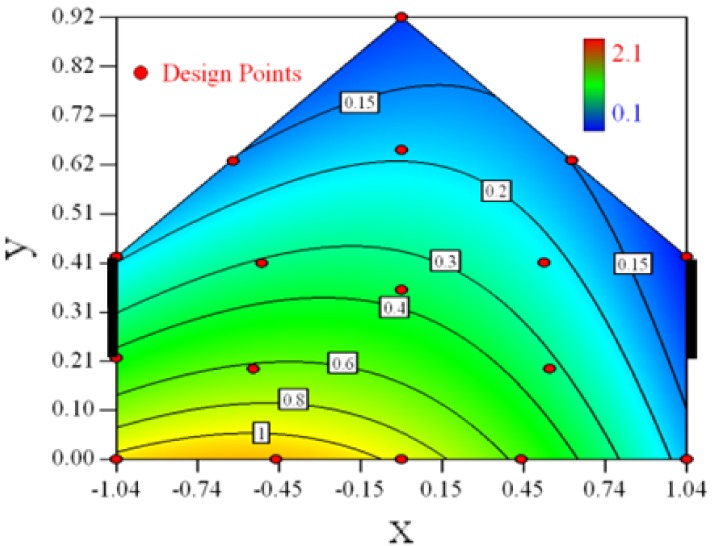

**Table 6.** Contours of the response suface of the desirability function for different experiemntal cases (Cases 1–8). The black bars in the plots represent the obstacles in the sidewall openings.In the plot, the incoming wind blows from the right side to the left.

**Response suface of the desirability function in Cases 1–8**			
Case 1	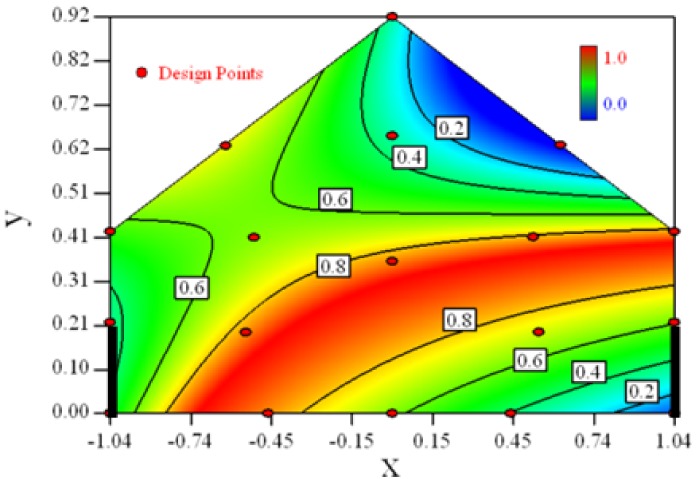	Case 7	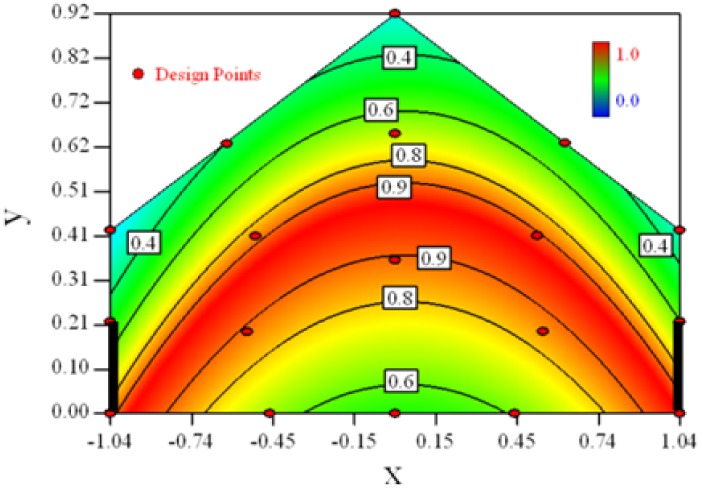
Case 2	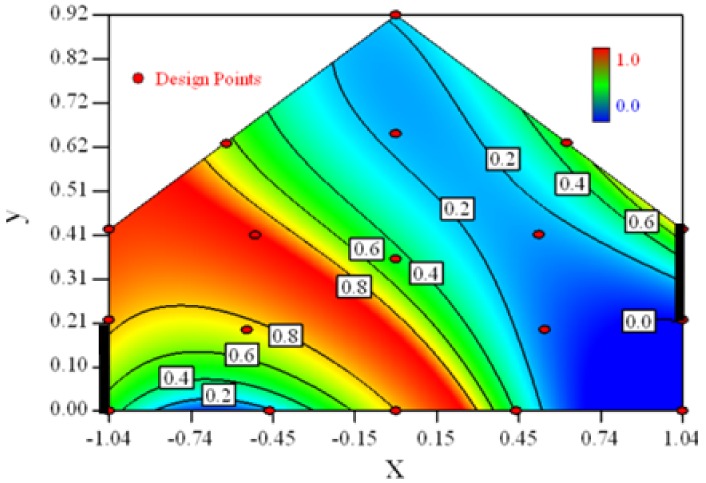	Case 8	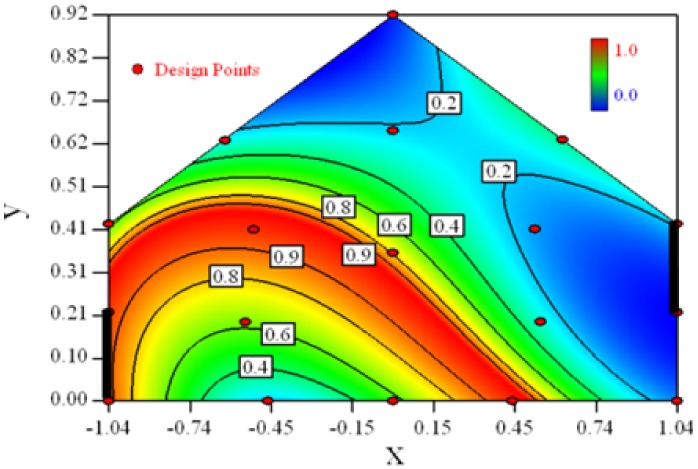
Case 3	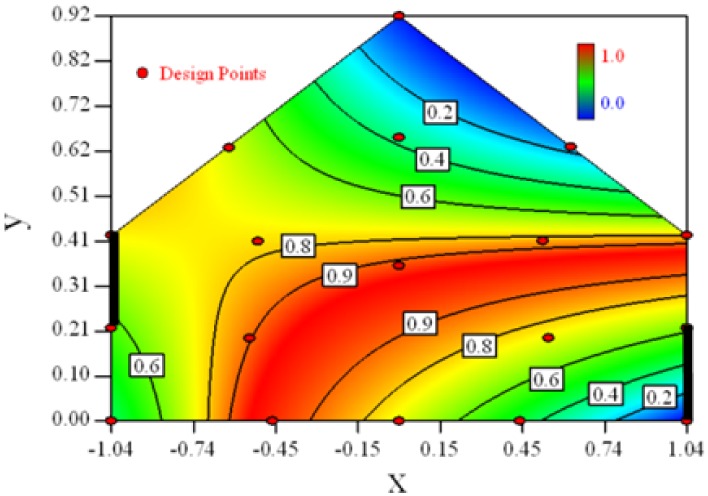	Case 6	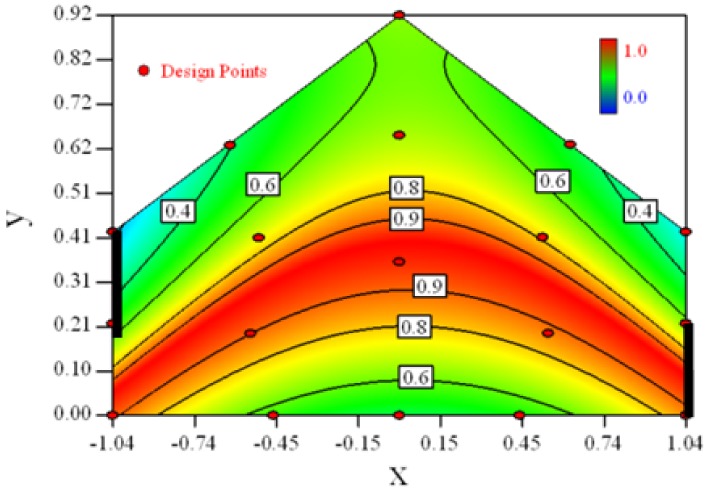
Case 4	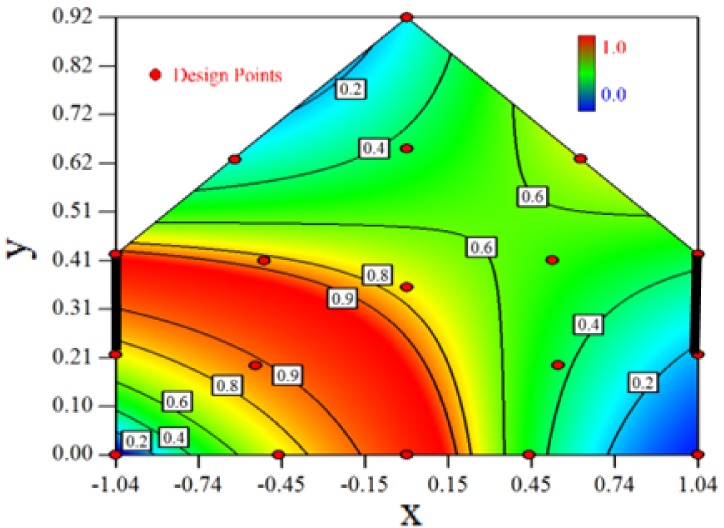	Case 5	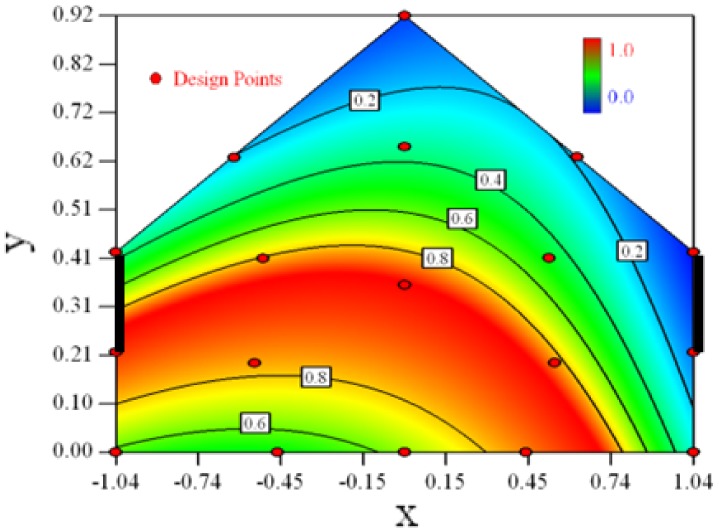

**Table 7.** Confirmation of the optimal sampling position (*x* = −*0.2, y* = *0.27*). The Estimated concentration ratio in the optimal position is calculated by the established RSM model of each case (Table 4). Surface averaged concentration ratio of each case is calculated from the RSM model (Table 4) by Equation 12; the estimated and actual desirability in the optimal position of an individual case (Cases 1–8) is calculated by Equation (11) from estimated and measured concentration ratio, respectively. The integrated desirability of all cases is calculated by Equation (10). The target is the surface averaged value of each individual case.

**Case**	**Estimated concentration ratio**	**Surface average**	**Estimated desirability**	**Measured concentration ratio**	**Actual desirability**
**1**	0.53	0.56	0.95	0.43	0.74
**2**	0.25	0.21	0.97	0.41	0.84
**3**	0.53	0.53	1.00	0.42	0.76
**4**	**0.15**	**0.14**	**0.99**	**0.07**	**0.40**
**5**	0.49	0.36	0.92	0.37	0.99
**6**	0.94	0.76	0.91	0.70	0.91
**7**	1.24	0.91	0.85	0.76	0.82
**8**	0.79	0.50	0.86	0.62	0.94
All			0.93		0.80
